# Supplementary material for: Calcium binding to a remote site can replace magnesium as cofactor for mitochondrial Hsp90 (TRAP1) ATPase activity
Source: J Biol Chem. 2018 Jul 10;293(35):13717–24. doi: 10.1074/jbc.RA118.003562 (PMC6120219; doi:10.1074/jbc.RA118.003562)
Supplement: Supporting Information [file supp_293_35_13717__index.html]

Calcium binding to a remote site can replace magnesium as cofactor for mitochondrial Hsp90 (TRAP1) ATPase activity — Modulation of TRAP1 ATPase activity by calcium and magnesium — Calcium binding to a remote site can replace magnesium as cofactor for mitochondrial Hsp90 (TRAP1) ATPase activity — Modulation of TRAP1 ATPase activity by calcium and magnesium — Supporting Information 

# Calcium binding to a remote site can replace magnesium as cofactor for mitochondrial Hsp90 (TRAP1) ATPase activity

## Supporting Information

- Supplemental Figure 1 - A comparison between a Michaelis-Menten fit versus the two-population model to ATPase activities of human TRAP1 in presence of MgCl2.
- Supplemental Figure 2 - ATPase activity of zebrafish TRAP1 in presence of MgCl2 or CaCl2.
- Supplemental Figure 3 - ATPase activities of Hsp90 homologs in presence of magnesium or calcium.
- Supplemental Figure 4 - Divalent cation titration versus ATPase activity in zebrafish TRAP1.
- Supplemental Figure 5 - Structures of zTRAP1 closed with AMPPNP.
- Supplemental Figure 6 - ATPase activity of hTRAP1 in presence of mixtures of magnesium and calcium.
- Movie 1 - Morph showing conformational changes in the ATP lid between apo and closed states as shown in Figure 3. The lid region (pink highlight) adopts an open state in absence of nucleotide (apo). The oxygen atoms involved in calcium binding (red spheres) do not form a cluster in the apo state. Once ATP binds the NTD, closing of the ATP-lid rearranges these oxygen atoms into a cluster.
