# Supplementary material for: Calcium binding to a remote site can replace magnesium as cofactor for mitochondrial Hsp90 (TRAP1) ATPase activity
Source: J Biol Chem. 2018 Jul 10;293(35):13717–24. doi: 10.1074/jbc.RA118.003562 (PMC6120219; doi:10.1074/jbc.RA118.003562)
Supplement: Supporting Information [file supp_RA118.003562_137575_2_supp_164338_pblz27.docx]

**Supplemental Figure 1**

**
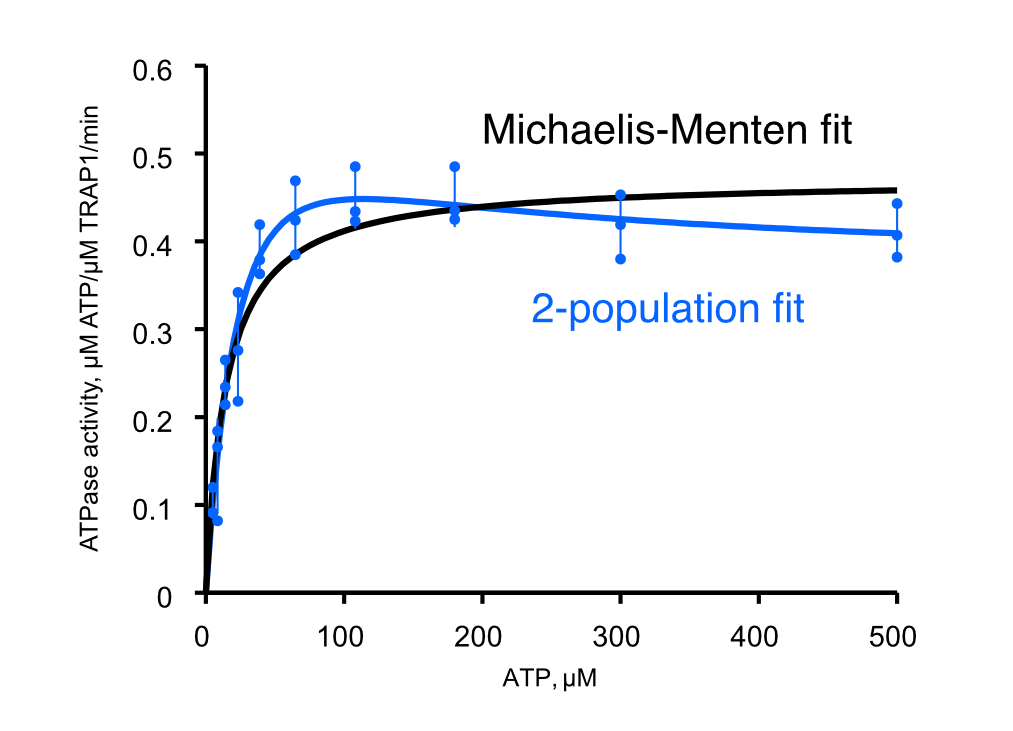
**

**Supplemental Figure 1.** A comparison between a Michaelis-Menten fit (black line) versus the two-population model (blue line) to ATPase activities of human TRAP1 in presence of MgCl_2_ (blue circles). Error bars are standard deviations from the data points shown as a scatter plot.
