# Supplementary material for: Calcium binding to a remote site can replace magnesium as cofactor for mitochondrial Hsp90 (TRAP1) ATPase activity
Source: J Biol Chem. 2018 Jul 10;293(35):13717–24. doi: 10.1074/jbc.RA118.003562 (PMC6120219; doi:10.1074/jbc.RA118.003562)
Supplement: Supporting Information [file supp_RA118.003562_137575_2_supp_164348_pb9z29.docx]

**Supplemental Figure 2**

**
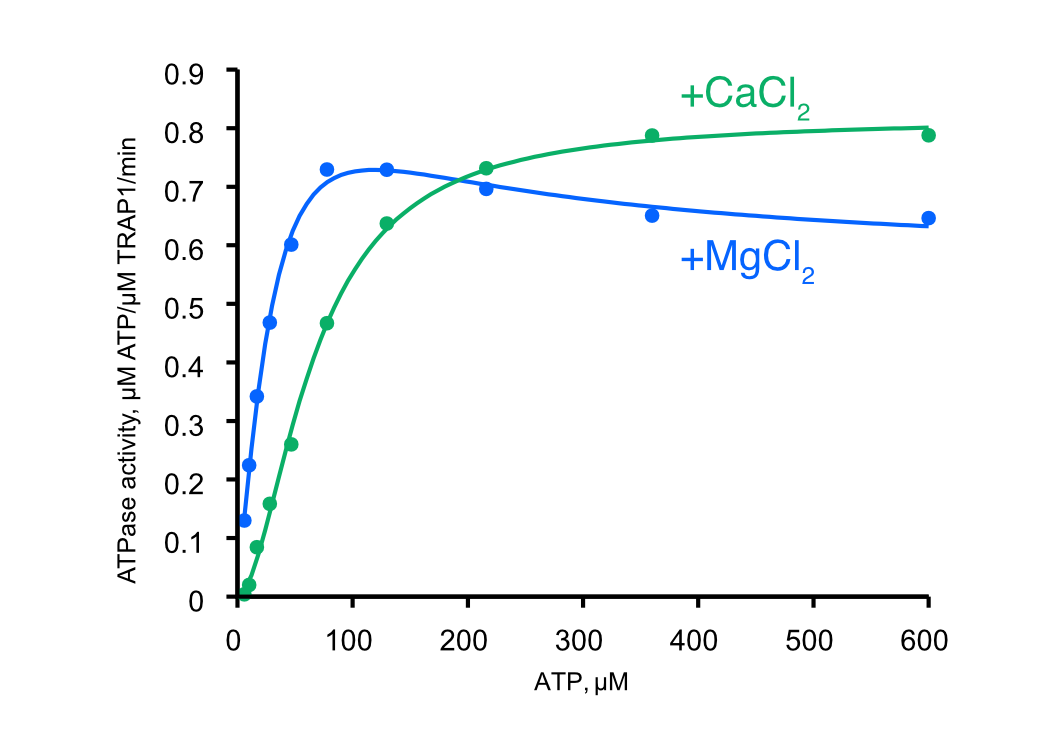
**

**Supplemental Figure 2.** ATPase activity of zebrafish TRAP1 in presence of MgCl_2_ or CaCl_2_. Steady-state ATPase activity with MgCl_2_ (blue) and CaCl_2_ (green) are shown as a function of ATP concentration at 30˚C.
