# Supplementary material for: Calcium binding to a remote site can replace magnesium as cofactor for mitochondrial Hsp90 (TRAP1) ATPase activity
Source: J Biol Chem. 2018 Jul 10;293(35):13717–24. doi: 10.1074/jbc.RA118.003562 (PMC6120219; doi:10.1074/jbc.RA118.003562)
Supplement: Supporting Information [file supp_RA118.003562_137575_2_supp_164349_pb5z29.docx]

**Supplemental Figure 3**

**
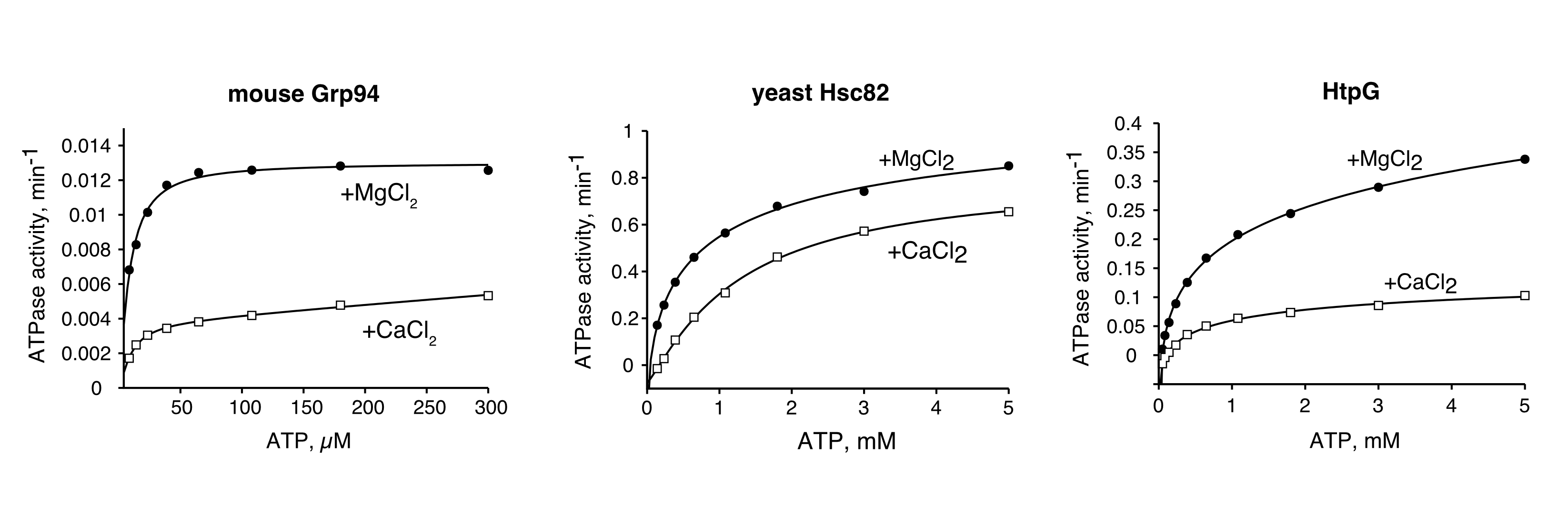
**

**Supplemental Figure 3.** ATPase activities of Hsp90 homologs in presence of magnesium or calcium. Steady-state ATPase assays as a function of ATP concentration is shown in presence of magnesium (filled black circles) or calcium (empty black squares). Full-length mouse Grp94 (left panel) at 25˚C, yeast Hsc82 (middle panel) at 30˚C, and HtpG (right panel) at 30˚C. For all experiments, the concentration of divalent cation is kept constant at 1 mM Grp94 and 5 mM for Hsc82 and HtpG.
