# Supplementary material for: Calcium binding to a remote site can replace magnesium as cofactor for mitochondrial Hsp90 (TRAP1) ATPase activity
Source: J Biol Chem. 2018 Jul 10;293(35):13717–24. doi: 10.1074/jbc.RA118.003562 (PMC6120219; doi:10.1074/jbc.RA118.003562)
Supplement: Supporting Information [file supp_RA118.003562_137575_2_supp_164346_pbpz29.docx]

**Supplemental Figure 4**

**
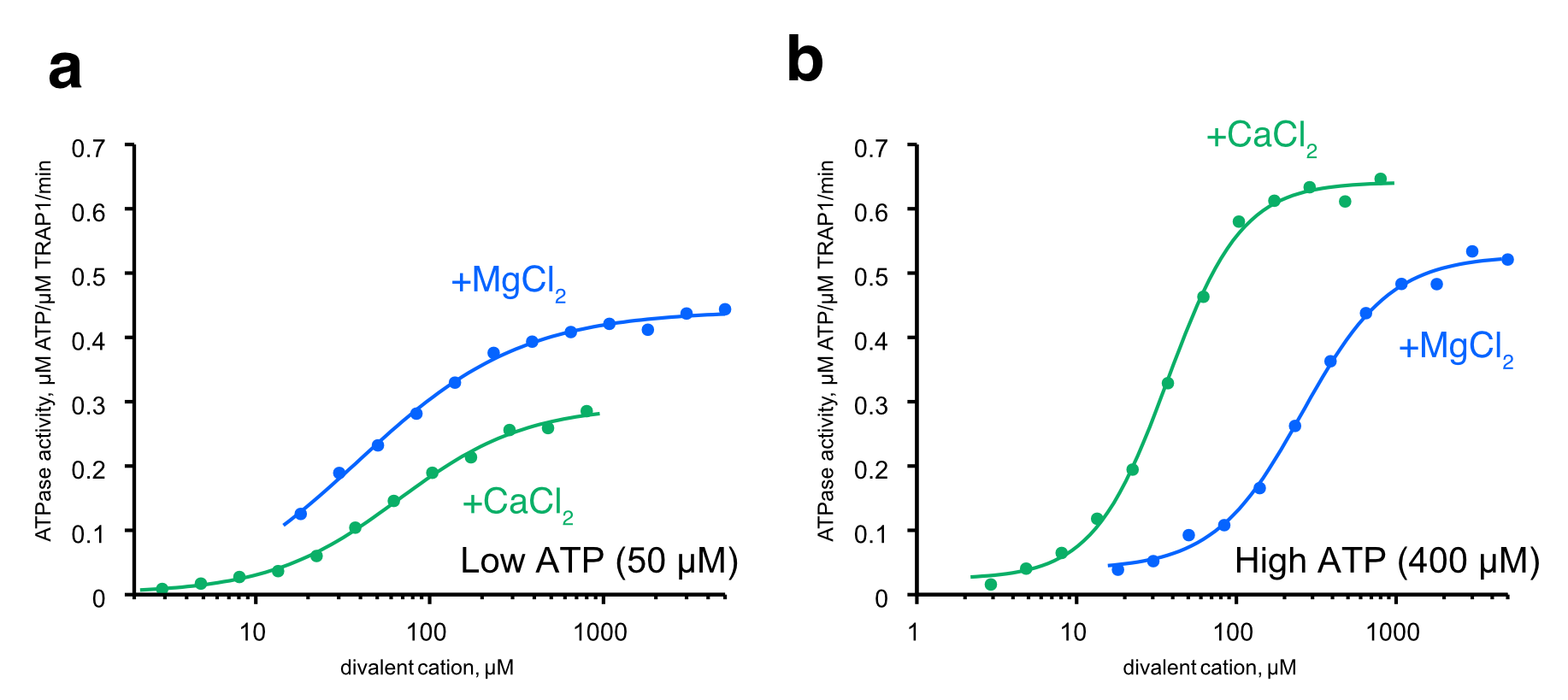
**

**Supplemental Figure 4.** Divalent cation titration versus ATPase activity in zebrafish TRAP1. **A**) ATPase activity of zebrafish TRAP1 in low (50 µM) ATP concentration. **B**) ATPase activity in high (400 µM) ATP concentrations. The same behavior is observed as in the same experiments with human TRAP1 in Figure 1E and 1F.
