# Supplementary material for: Calcium binding to a remote site can replace magnesium as cofactor for mitochondrial Hsp90 (TRAP1) ATPase activity
Source: J Biol Chem. 2018 Jul 10;293(35):13717–24. doi: 10.1074/jbc.RA118.003562 (PMC6120219; doi:10.1074/jbc.RA118.003562)
Supplement: Supporting Information [file supp_RA118.003562_137575_2_supp_164337_pb3z27.docx]

**Supplemental Figure 5**

**
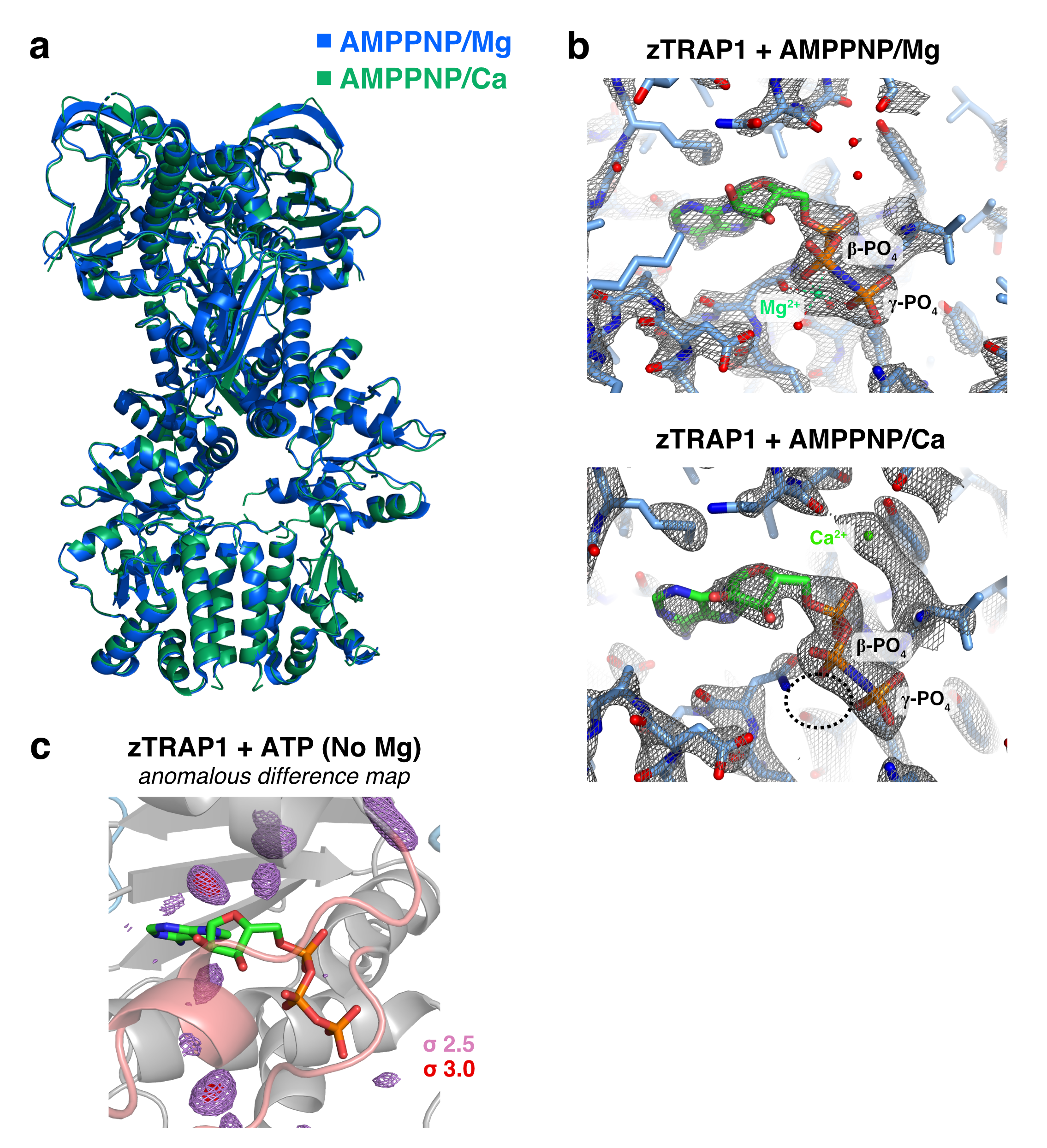
**

**Supplemental Figure 5.** Structures of zTRAP1 closed with AMPPNP. **a**) Superimposed structures of zTRAP1 with AMPPNP/Mg (Protein Data Bank code 4IPE) (blue cartoon) and AMPPNP/Ca (Protein Data Bank code 6D14, release pending) (green cartoon) showing identical closed states with an rmsd of 0.585 Å. **b**) Electron density (2Fo-Fc) maps (gray mesh) around AMPPNP of zTRAP1 closed with AMPPNP/Mg (top panel) showing magnesium (lime-green sphere) coordinated between the β- and γ-phosphates. For comparison, zTRAP1 closed with AMPPNP/Ca (bottom panel) is missing electron density in the same region (dashed ellipse). Both density maps are shown at 2.8σ contour level. **c**) Anomalous difference maps at various contour levels, σ, 2.5 (pink), and 3.0 (red) displayed in mesh of zTRAP1 closed with ATP and excess EDTA. At lower contour (σ2.5) the maps are noisy and at no strong density is above the α-phosphate is visible at σ3.0.
