# Supplementary material for: Calcium binding to a remote site can replace magnesium as cofactor for mitochondrial Hsp90 (TRAP1) ATPase activity
Source: J Biol Chem. 2018 Jul 10;293(35):13717–24. doi: 10.1074/jbc.RA118.003562 (PMC6120219; doi:10.1074/jbc.RA118.003562)
Supplement: Supporting Information [file supp_RA118.003562_137575_2_supp_164350_pbsz29.docx]

**Supplemental Figure 6**


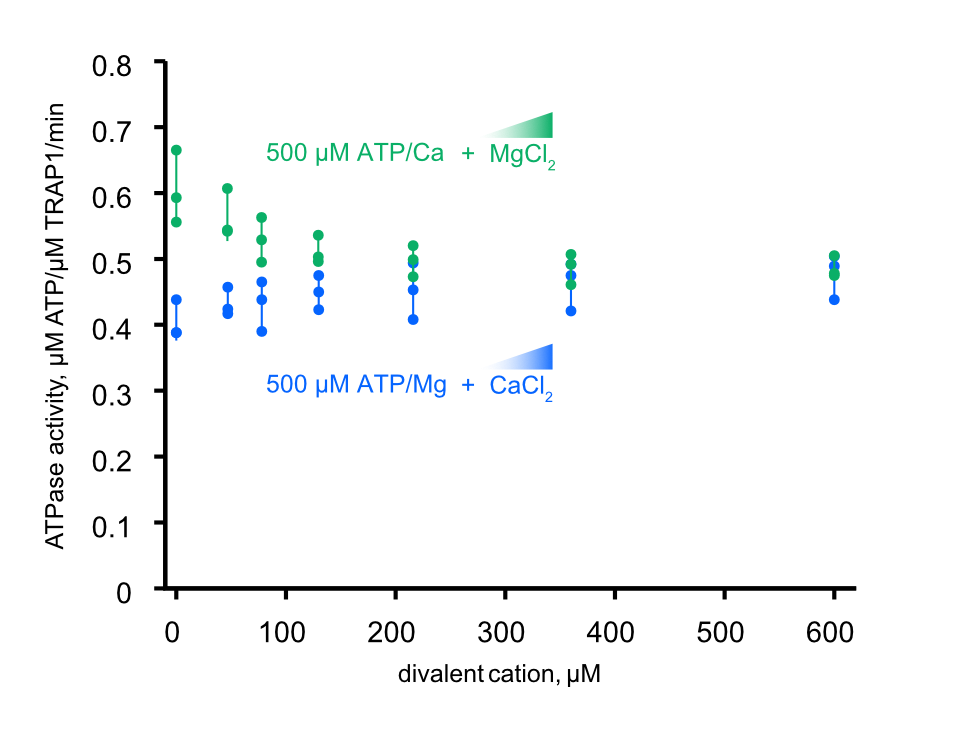


**Supplementary Figure 6.** ATPase activity of hTRAP1 in presence of mixtures of magnesium and calcium. The concentration of ATP/Mg is held fixed starting at 500 µM while titrating CaCl_2_ up to 600 µM (blue). The concentration of ATP/Ca is held fixed while titration MgCl2 up to 600 µM (green). The ATPase activity for the magnesium/calcium mixture converges to roughly the average value of the initial points where only one species of divalent cation is present. Error bars are standard deviations calculated from the data points shown as scatter plots.
